# Dynamics of viral replication in blood and lymphoid tissues during SIVmac251 infection of macaques

**DOI:** 10.1186/1742-4690-6-106

**Published:** 2009-11-23

**Authors:** Abdelkrim Mannioui, Olivier Bourry, Pierre Sellier, Benoit Delache, Patricia Brochard, Thibault Andrieu, Bruno Vaslin, Ingrid Karlsson, Pierre Roques, Roger Le Grand

**Affiliations:** 1 CEA, Division of Immuno-Virology, DSV/iMETI, Fontenay-aux-Roses, France; 2 Université Paris-Sud 11, UMR E01, Orsay, France; 3 Assistance Publique-Hôpitaux de Paris, Service de Médecine Interne A, Hôpital Lariboisière, France

## Abstract

**Background:**

Extensive studies of primary infection are crucial to our understanding of the course of HIV disease. In SIV-infected macaques, a model closely mimicking HIV pathogenesis, we used a combination of three markers -- viral RNA, 2LTR circles and viral DNA -- to evaluate viral replication and dissemination simultaneously in blood, secondary lymphoid tissues, and the gut during primary and chronic infections. Subsequent viral compartmentalization in the main target cells of the virus in peripheral blood during the chronic phase of infection was evaluated by cell sorting and viral quantification with the three markers studied.

**Results:**

The evolutions of viral RNA, 2LTR circles and DNA levels were correlated in a given tissue during primary and early chronic infection. The decrease in plasma viral load principally reflects a large decrease in viral replication in gut-associated lymphoid tissue (GALT), with viral RNA and DNA levels remaining stable in the spleen and peripheral lymph nodes. Later, during chronic infection, a progressive depletion of central memory CD4+ T cells from the peripheral blood was observed, accompanied by high levels of viral replication in the cells of this subtype. The virus was also found to replicate at this point in the infection in naive CD4+ T cells. Viral RNA was frequently detected in monocytes, but no SIV replication appeared to occur in these cells, as no viral DNA or 2LTR circles were detected.

**Conclusion:**

We demonstrated the persistence of viral replication and dissemination, mostly in secondary lymphoid tissues, during primary and early chronic infection. During chronic infection, the central memory CD4+ T cells were the major site of viral replication in peripheral blood, but viral replication also occurred in naive CD4+ T cells. The role of monocytes seemed to be limited to carrying the virus as a cargo because there was an observed lack of replication in these cells. These data may have important implications for the targeting of HIV treatment to these diverse compartments.

## Background

Viral RNA quantification in plasma provides important insight into the natural course of HIV infection and is widely used in both acute and chronic infection as a surrogate marker for the evaluation of disease progression [1,2]. Other markers such as viral DNA in peripheral blood mononuclear cells (PBMC) have been used to predict disease progression from primary infection [3,4]. The simultaneous determination of viral RNA in plasma and viral DNA in PBMCs has been shown to be more robustly related to clinical outcome [3,5]. These studies highlight the importance of evaluating events occurring during primary infection to improve our understanding of HIV pathogenesis.

It is difficult to study primary infection in humans, particularly those that concern the dynamics of viral infection in deep tissues. Non-human primate models of HIV infection are therefore of particular importance. Only a few studies have focused on these aspects. Mattapallil *et al. *demonstrated, by quantifying SIV-gag DNA, that the high levels of free virus in plasma at the peak of primary SIV infection are associated with maximal viral spread and high rates of viral replication in all lymphoid tissues [6]. Other studies have reported viral replication in gut-associated lymphoid tissue (GALT). Li *et al. *showed that the levels of SIV mRNA in the GALT of SIV-infected macaques decreased by a factor of 20 between peak plasma viral load (PVL) and day 28 post infection (pi) [7]. The high levels of viral replication in GALT at peak infection resulted in a profound depletion of CD4+ T lymphocytes, which could potentially lead to the immunodeficiency observed in the long term. However, these studies addressed only the short-term dynamics of viral replication in tissues with a maximum follow-up of 28 days pi. The studies used only RNA or total DNA viral markers. Viral RNA has classically been used to evaluate viral replication or production, whereas viral DNA is generally used to evaluate dissemination.

The 2LTR circular viral DNA is another viral marker. It is an extrachromosomal product formed after the entry of the virus into the target cell and following its reverse transcription. This structure results from the circularization of two long terminal repeats of linear viral DNA by cellular DNA repair factors [8,9] in the absence of integration. Despite the fact that contradictory studies have been reported [10-13], the 2LTR circles are labile in vivo and may therefore be used as an indicator of recently infected cells [14].

We used cynomolgus macaques infected with SIVmac251 to study in detail the dynamics of viral replication in peripheral blood and tissues during primary and early chronic infection as well as its impact in the long term. We studied both free virus levels in plasma and viral replication in lymphoid tissues from peak PVL to the set point, both of which were two key dates for predicting the rate of disease progression in the long term. We used a combination of three viral markers simultaneously to study in detail viral dissemination and the dynamics of viral replication in tissues: viral DNA (indicating dissemination), viral RNA (an indicator of viral replication and production), and 2LTR circles (to identify recently infected cells) [12,14-17].

## Results

### Determinations of viral RNA in plasma and of viral DNA and 2LTR circles in PBMCs at the set point may predict the long-term progression of SIV infection

**Figure 1:**
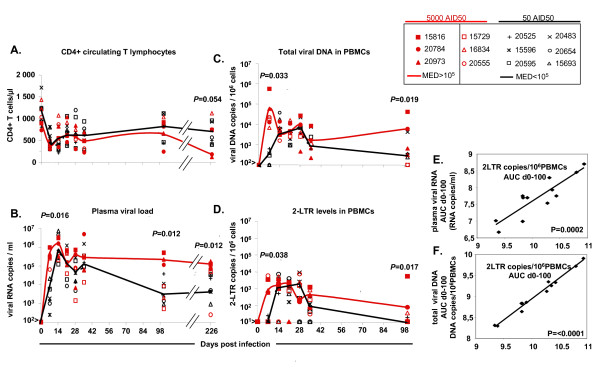
**The dynamics of CD4+ T cells, viral replication and dissemination of the virus in the peripheral blood of SIV-infected macaques**. We divided macaques into the low and high replication groups (black and red full lines, respectively), regardless of the viral doses used for inoculation, and according to the level of plasma viral load at set point (day 100 pi. 10^5^/ml copies RNA). The symbols of macaques infected with low dose (50 AID50) and high dose (5,000 AID50) were represented by black and red colors respectively. (A) Changes in absolute CD4+ T-cell counts in peripheral blood. (B-C-D) Changes in viral RNA levels in plasma and viral DNA and 2LTR circle levels in the PBMCs. (E-F) Correlations between 2LTR circle levels and viral DNA or plasma viral RNA levels.

We and others have previously evaluated the relevance of viral RNA determinations in plasma for predicting disease progression [18]. We monitored plasma viral RNA (vRNA), total viral DNA (vDNA), and 2-LTR circle levels in parallel in PBMCs from cynomolgus macaques inoculated intravenously with SIVmac251 (Figure 1) for a more precise characterization of viral dynamics during the first few weeks of primary infection. We have demonstrated that this virus is pathogenic in this species, and different profiles of viral and immunological parameters could be identified depending on the dose and route of inoculum [18-21].

We intravenously injected two groups of six macaques each with a high dose (5,000 AID50) or a low dose (50 AID50) of pathogenic SIVmac251 in order to generate different disease progression profiles. These infections generated two different profiles in terms of vRNA levels at set point (day 100 pi): a group of rapidly progressing animals with high plasma viral load (>10^5 ^vRNA copies/ml) and a group of moderately progressing animals with a significantly lower (p = 0.012) plasma viral load (<10^5 ^vRNA copies/ml). This pattern was confirmed in the long term, on day 226 pi, with plasma viral load continuing to exceed 10^5 ^vRNA copies/ml and a significant decrease in CD4 counts (p = 0.054; CD4+ = 324 ± 373) in the highly viraemic group. The animals in the group with less than 10^5 ^vRNA copies/ml displayed slower disease progression as demonstrated by the maintenance of high levels of CD4 counts (CD4+ = 719 ± 281) (Figure 1). These data are consistent with published data from our group and other groups working on the same SIV-macaque model [18,22,23].

MHC typing from individual animals of groups 5000 and 50 AID50 were performed and showed a relative homogeneity of haplotype class II. One animal of the progressor group and two animals from 50 AID were haplotype H6 (data not shown) which is known to be associated with low disease progression [24].

We investigated viral dissemination in the groups displaying rapid and moderate progression by following the dynamics of viral DNA and 2LTR circles in PBMCs. At the set point, as for vRNA in plasma, viral DNA and 2LTR circle levels in PBMC were significantly higher in the rapid progression group (0.019 and 0.017 respectively) than in the moderate progression group. Moreover, all the viral parameters determined in peripheral blood (vRNA in plasma, vDNA and 2LTR circles in PBMCs) increased significantly earlier (day 7 pi) in the rapid progression group than that in the moderate progression group (p = 0.016, p = 0.033, p = 0.038, respectively) (Figure 1B-D). Thus, our results confirm that the early spread and persistence of high levels of viral replication in peripheral blood during primary infection may predict rapid disease progression.

There was a significant, strong correlation between plasma viral RNA levels and the levels of viral DNA or 2LTR circles in PBMCs during infection (day 0 to 100 pi.), as determined by measuring the area under the curve (Spearman's rank correlation test, p ≤ 0.0002 and p ≤ 0.0001, respectively) (Figure 1E-F). Thus, during this period, viral DNA and 2LTR circle levels in PBMC changed in the same manner as plasma viral RNA levels.

### Plasma viral load is correlated with viral replication in gut-associated lymphoid tissue during SIVmac251 primary infection in macaques

We extended this analysis to tissues to improve our understanding of the relationship between the kinetics of viral replication in blood and viral dissemination in tissues at peak of viremia and at the set point. We focused our analysis on the tissues thought to be the main sites of viral replication, such as digestive tract (ileum and rectum) and secondary lymphoid (spleen, peripheral and mesenteric LN) tissues.

**Figure 2:**
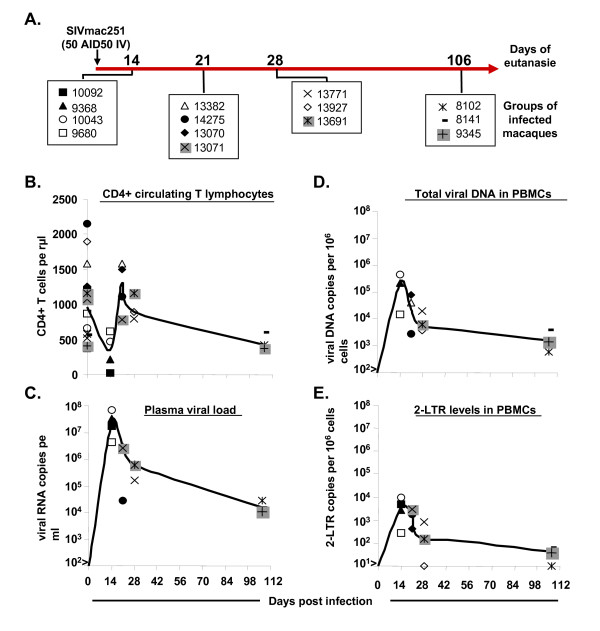
**Changes in CD4+ T cell numbers as a function of viral replication and dissemination in the peripheral blood, in four groups of SIV-infected macaques during primary infection**. (A) Protocol for SIV infection, evaluations, and the euthanasia of each animal. Each box indicates the group of macaques explored at the corresponding times. (B) Changes in absolute counts of total CD4+ T cells in peripheral blood. (C-D-E) Changes in viral RNA levels in plasma and viral DNA and 2LTR circle levels in PBMCs. Bold lines indicate the mean value (B-D-C-E).

Another group of fourteen macaques were infected with 50 AID_50 _of the same SIVmac251 viral stock. As expected, they showed a pattern of moderate progression involving a slow decrease in CD4 counts and PVL similar to that observed in the majority of humans infected with HIV-1. The animals were then euthanized, on day 14 (4 animals), 21 (4 animals), 28 (3 animals) or 100 (3 animals) pi (Figure 2A). For each animal, we simultaneously analysed viral RNA levels in plasma and tissue and total viral DNA and 2-LTR circle levels in both PBMC and tissues.

The immunological and virological patterns in peripheral blood of these animals (Figure 2B-E) were similar (similar curves for CD4+T-cell counts, plasma viral RNA, total DNA and 2LTR circle levels) to that we previously reported for macaques receiving the same dose of virus.

**Figure 3:**
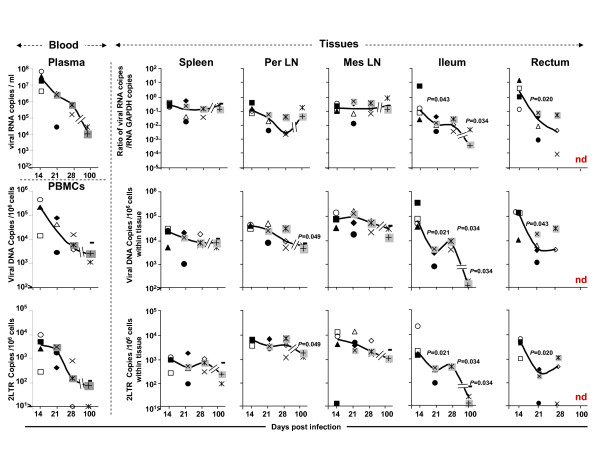
**Viral replication and dissemination in the tissues of macaques during primary infection with SIVmac251**. The three viral markers -- viral RNA, DNA and 2 LTR circles -- were evaluated in various tissues from macaques infected with SIVmac251, on days 14, 21, 28 and 100 pi. The relative level of viral RNA with respect to the mRNA for GAPDH was calculated by the "delta delta Ct" method. Absolute copy numbers for viral DNA and 2LTR circles were calculated to the GAPDH and normalized to one million of cells. When significant, p values were indicated. The results from blood were added to tissues as comparative value.

An analysis of viral RNA levels in plasma and tissues on day 14 pi showed that peak plasma viral load was associated with a very high level of viral replication in all the tissues explored (Figure 3). Parallel evaluations of both viral DNA and 2LTR circles in PBMCs and tissues showed that the cell-associated viral load peak in PBMCs was also accompanied by high levels of viral dissemination in all tissues (Figure 3). At this time point, no major difference in the level of viral replication or dissemination was observed between the different tissues (Figure 3). Thus, at peak viraemia, viral replication and dissemination levels were maximal in all lymphoid tissues. On day 21 post infection, when plasma viral load began to decrease, we observed a significant decrease in SIV RNA level in the GALT, whereas SIV RNA levels remained stable in the spleen and peripheral lymph nodes. The decrease in SIV RNA levels in the GALT was associated with decreases in the levels of both SIV DNA and 2LTR circles in this tissue (Figure 3). We assumed, as previously reported for this model, that the simultaneous decrease in all three markers would result from the loss of infected cells in this compartment [25].

Plasma viral load was slightly lower on day 28 than on day 21 pi, but viral RNA levels in all lymphoid tissues remained roughly constant. Viral DNA and 2LTR circle levels in PBMCs displayed a similar pattern (Figure 3).

By the set point, on day 100 pi, plasma RNA load was significantly lower than on day 28 pi, and we observed small numbers of infected cells and low levels of viral replication in the GALT, as demonstrated by the parallel decreases observed in SIV RNA/DNA and 2LTR circle levels in this compartment (Figure 3).

**Figure 4:**
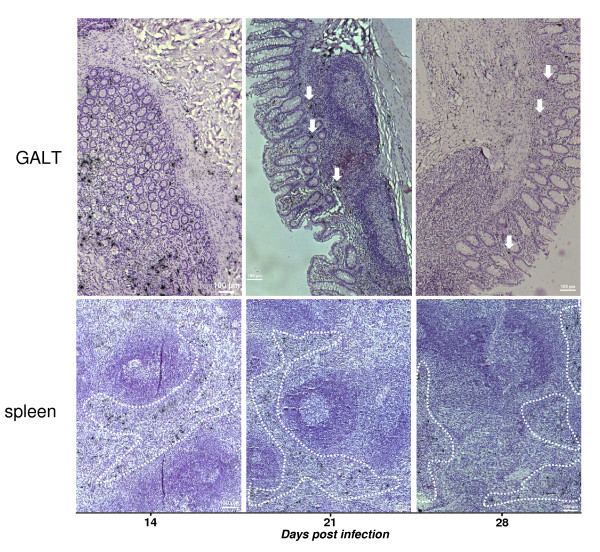
**Viral transcription in the two examples of tissue, GALT and spleen, at 14, 21 and 28 days pi**. In situ hybridization was performed with radiolabeled SIV-specific RNA and SIV RNA-positive cells appear black. Montage of large image (magnification ×10) of single section, among 4 or 2 sections examined from GALT and spleen, respectively. The encircled regions in the spleen were the most numerous for productive cells. The headed arrow points to a few SIV RNA positive cells founded at day 21 and 28 in the GALT.

The analysis of viral RNA in the tissues by PCR was enhanced by in situ hybridisation assays. We confirmed that at day 14 dense collections of SIV RNA-positive cells developed in the GALT and the spleen. The SIV RNA-positive cells decreased from day 21 to 28 in the GALT, whereas they were still detectable in the spleen (Figure 4). A qualitative assessment revealed at day 14 pi, that SIV RNA-positive cells were detected in the GALT with no preferential localization (such cells were detected in the germinal centers as well as in the lamina propria), thereafter the SIV RNA-positive cells became localized mainly in the lamina propria., SIV RNA-positive cells in the spleen were essentially localized around germinal centers and in the white pulp regardless of the date of infection (Figure 4).

**Figure 5:**
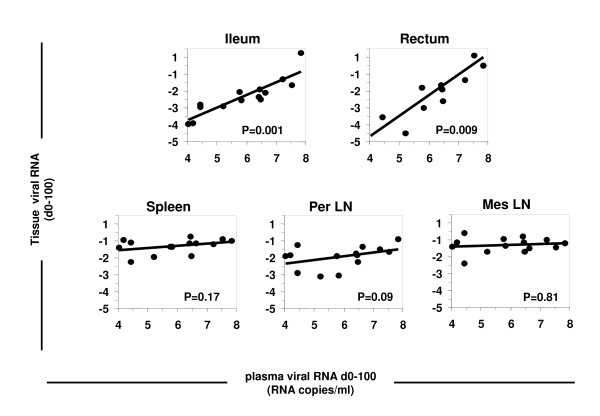
**Correlation between plasma viral load and SIV RNA level in the GALT and the secondary lymphoid tissue from 0 to 100 days pi**.

Because we observed parallel decreases in the number of infected cells/level of viral replication in the GALT and plasma viral load during primary infection with SIV, we hypothesized that the GALT was the principal source of the virus in the plasma. We tested this hypothesis by assessing the correlation between viral production in each tissue and plasma viral load during primary infection with SIV. As expected, we found a very strong correlation between SIV RNA level in the ileum or rectum and plasma viral load (p = 0.0097 and p = 0.001, respectively) but no correlation with viral load in other lymphoid tissues (spleen: p = 0.17, peripheral LN: p = 0.097, mesenteric LN: p = 0.81) could be established (Figure 5).

### Levels of viral replication in peripheral blood during chronic infection differ considerably between central memory CD4+ T cells, naive CD4+ T cells and monocytes

We assessed the effect of viral load during primary infection on subsequent virus progression during the chronic phase of infection. We chose six macaques from the moderate progression group (with viral loads <10^5 ^copies RNA/ml at set point). After two years of infection, we investigated changes in viral and immunological parameters in the peripheral blood. At that time, the macaques had slightly higher plasma viral loads (mean = 3.7 ± 0.6, 100 days pi vs. 4.5 ± 0.4, 2 years pi.) and a markedly higher cell-associated viral load (viral DNA mean = 2.6 ± 0.5, 100 days pi vs. 3.7 ± 0.3, 2 years pi; 2LTR circles mean = 1.0 ± 0.1, 100 days pi vs. 2.2 ± 1.1, 2 years pi) when compared to viral load at the set point. The proportion of circulating CD4+ T cells and particularly of CD4+ central memory lymphocytes was also lower (38 ± 6%, 100 days pi vs. 15 ± 5%, 2 years pi.).

**Figure 6:**
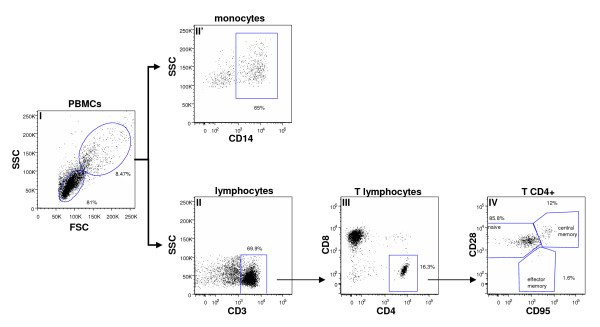
**Flow cytometric sorting strategy for monocytes and T cells**. A representative exemple is shown. PBMCs from each animal were stained with the antibody combination described in the material and methods. Monocytes and lymphocytes were defined with forward and side scatter (I). CD3+ T cells were then defined based on expression of CD3 (II). CD4+ T cells were then defined based on expression of CD4 without expression of CD8 (III). Naïve CD4+ T cells were then separated based on expression of CD28 without expression of CD95 (IV). Central memory CD4+T cells were then separated based on dual expression of CD28 and CD95 (IV). Effector memory CD4+ T cells were then separated based on expression of CD95 without expression of CD28 (IV). The CD14+ monocytes were separated based on expression of CD14+ (II').

We therefore tried to identify the infected peripheral cells in which active replication of the virus occurred. We sorted naive lymphocytes (CD4+CD28^high^CD95^low^), central memory lymphocytes (CD4+CD28^high^CD95^high^), effector memory (CD4+CD28^low ^CD95^high^) lymphocytes and CD14+ monocytes (Figure 6), with a mean purity higher than 96% (Table 1). In each cell subset we quantified viral RNA, total viral DNA, and 2LTR circles.

**Table 1:** Purity of sorted T cells and monocytes

T CD4+ lymphocytes				
**sample**	**Naive** **CD28^high^CD95^low^**	**central memory** **CD28^high^CD95^high^**	**effector memory** **CD28^lox^CD95^high^**	**CD14+ monocytes**

15596	99	97	96	97

15693	98	98	97	98

20483	97	96	95	98

20525	99	97	96	99

20595	98	98	95	98

20654	96	99	97	98

Mean ± SEM	98.0 ± 1.2	98.0 ± 1.0	96.0 ± 0.9	98.0 ± 0.6

**Figure 7:**
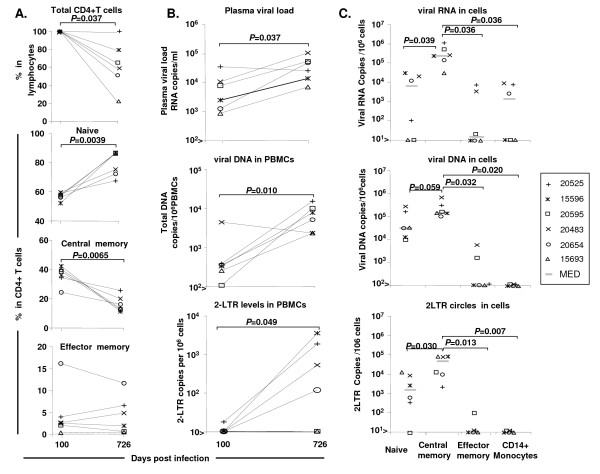
**Changes in immunological parameters and compartmentalisation of the virus in various cell subtypes in the peripheral blood during the chronic phase of infection**. (A) Changes in the total number of CD4+ T cells and of their various subtypes, such as naive, central memory and effector memory cells, in the peripheral blood between set point on day 100 pi and 2 years pi. (B) Changes in plasma viral RNA, viral DNA, and 2LTR circle levels in PBMCs between set point on day 100 pi and 2 years pi. (C) Distribution of viral RNA, viral DNA and 2LTR circles in naive, central memory and effector memory lymphocyte subsets and in CD14+ monocytes from PBMCs, during chronic infection. The cell sorting was performed twice from each animal and each RT-PCR or PCR was quantified in duplicate.

Both central memory CD4+ T cells and naive cells were involved in viral dissemination, but the total viral DNA content of the central memory T cells (mean: 5.4 ± 0.3 viral DNA copies/10^6 ^cells) was 1 log higher than that of the naive cells. Effector memory cells contained little viral DNA, and monocytes had almost no viral DNA (Figure 7).

Central memory CD4+ T cells and naive cells were both involved in the viral infection/replication process despite the significantly lower SIV RNA levels in naive than in central memory cells. Viral DNA and RNA were nonetheless observed in the naive cell subsets of almost all the animals (5/6). Low levels of viral infection and replication were observed in cells of the effector memory subset in only two of the six animals. Unexpectedly, we detected SIV RNA in monocytes from three animals, despite the absence of SIV-DNA and 2LTR circle detection in this cell subset. Thus, central memory and naive CD4+ T cells may play a key role in both viral dissemination and viral replication (Figure 7).

## Discussion

In this study, we used a combination of three SIV markers to investigate viral dissemination and replication in peripheral blood and tissues: viral RNA, viral DNA, and 2-LTR circles. We found a linear correlation between plasma viral RNA levels and total viral DNA or 2-LTR circle levels in circulating PBMCs. Similar observations were reported for viral RNA and DNA loads during primary viremia in SIV infected cynomolgus macaques[26].

We also report here the first simultaneous determination of these three markers in the main lymphoid tissues including the GALT. For each tissue, we observed a significant correlation between the three viral markers (p = 0.0001). We also found no relevant differences in the ratio of 2LTR circle to total viral DNA levels in the different types of sample at any of the times studied, confirming the lack of accumulation of 2LTR circles. Thus, in each tissue, the three viral markers varied in the same manner, reflecting the level of viral replication.

We monitored viral load in the peripheral blood of SIVmac251-infected macaques for 226 days after infection. Our findings confirm that plasma vRNA load at set point is predictive of disease progression, as previously reported [23,27]. Our results also suggest that the combination of a rapid increase in viral load and the persistence of a high viral load until the set point in both plasma and PBMCs may distinguish macaques with rapid disease progression from those with intermediate progression. Thus, rapid viral spread may be critical for the establishment of persistent viral replication and may be associated with rapid disease progression [2,4,28,29].

The plasma viral load, and subsequent circulating CD4 depletion, principally reflected viral replication in the GALT during primary infection [30-32]. This relationship between peripheral blood viral load and replication in the GALT is not particularly surprising. Indeed, only 2% of circulating T lymphocytes are found in the peripheral blood [33], whereas the GALT contains most of the T lymphocytes in the body 40 to 60% [34,35]. In both humans [36-38] and macaques [6,39], most (> 95%) CD4+ T lymphocytes in the GALT are CD45RA- or activated memory T lymphocytes, and about 30 to 75% of these cells express CCR5 [40,41]. The GALT may therefore constitute a major site of viral replication, providing the peripheral blood with free virus. During primary infection, we observed a parallel decrease in vRNA levels in the GALT and plasma, probably due to the progressive depletion of activated memory CD4+ T cells during primary infection in this tissue [25]. Other compartments, including the PBMCs and lymph nodes, despite stable viral replication in the latter, may also supply the plasma with free virus, but probably to a lesser extent, due to their reduced size as compared to lymphoid compartment in mucosal tissues [34,35].

Activated memory CD4+ T cells are depleted from all lymphoid tissues early in infection [6]. However, the composition of CD4+ T lymphocytes subsets from lymph nodes is different from that in the GALT [6,42,43]. Lymph nodes contain larger numbers of resting memory CD4+ T lymphocytes which can be productively infected [7] but are probably more resistant to death, explaining the persistence of viral replication in the spleen and lymph nodes that we observed in our study [25,30].

As expected, we observed a slight increase in viral load in the peripheral blood and the depletion of central memory CD4+ T cells after two years of SIV infection. An extensive analysis of viral replication in peripheral cell subsets showed this subpopulation to be highly permissive to the virus and to be the principal location of viral RNA and DNA in the peripheral blood, consistent with previous findings [6,30]. These results also suggest that central memory CD4+ T cell depletion may be a consequence of the high levels of viral replication and activation in this cell subset. Viral replication was also detected in naive CD4+ T cells. Despite having viral loads ~100 fold lower than that of central memory CD4+ T lymphocytes, naive CD4+ T cells may be actively involved in viral replication, particularly as they account for 65 to 85% of all CD4+ T lymphocytes in peripheral blood. These results raise questions about the precise role of naive CD4+ T cells in viral replication *in vivo*. *In vitro *studies have generally assumed that naive CD4+ T cells are resistant to SIV/HIV infection, because they are in the G0 phase of the cell cycle and are not activated [44-46]. However, in many *in vivo *reports, naive cells have been shown to support infection [36,40,47]. The apparent conflict between *in vitro *resistance and *in vivo *susceptibility of naïve CD4+ T cells to viral replication could be explained by the role of the microenvironment as previously reported [48].

Alternatively, infected CD4+ T cells may be generated from infected thymocytes as suggested by our data (Additional File 1) and other reports [26]. In addition recent *ex vivo *data for humans have suggested that the R5 strain preferentially infects and replicates in mature CD3+/hi CD27+ thymocytes [49]. The thymus is essential for the initial seeding of T cells to the periphery and continues to produce naive T cells in middle-aged humans [50]. This would result in naive circulating CD4+ T cells replicating the virus and contributing to the dissemination of the virus when these cells migrate from the blood to other anatomic sites.

Some exceptions to the relationships between the studied viral parameters within the various cellular compartments were observed in the monocytes which contained low frequency viral RNA but had undetectable levels of vDNA and 2LTR circles (Figure 7C). Kaiser et al. have reported in untreated HIV patients the absence of vDNA and low frequencies of viral RNA in this cell subtype (100- to 1,000-fold lower than those of HIV-infected CD4+ T cells) [51]. Thus, monocytes appeared unlikely to play a major role for virus production in peripheral blood. However, it would be important in follow-up studies to look at tissue macrophages. On the other hand, the absence of viral RNA and 2LTR circles from the naïve CD4 T cells of animal 20595 despite the presence of viral DNA (Figure 7C) could be related to viral latency, although it was not clearly demonstrated in this cell subtype. Finally, effector cells were those reported with the strongest disparity (Figure 7C). These cells could contain only viral RNA (animal 20525), both viral RNA and DNA without 2LTR circles (animal 20483), slight detection of the three markers (20595), or lack of the viral markers (animals #20654 #15596 #15693). However, apparent discrepancies could be attributed to cells coated with virus without infection, cells infected with a very slowly replicating virus, or cells resistant to infection. CCR5 positive effector cells in blood and other tissues may however differ in differentiation stage and/or activation status, resulting in different capacity for viral replication.

Dynamics of viral replication in the acute phase could be different after intrarectal- or intravaginal transmission as compared to intravenous inoculation. Our preview studies after iv, intrarectal or intravaginal inoculation showed among other hypothesis, a delay of plasma viral load in early infection from the three routes of infection [19-21,52]. This delay could be explained by differences in virus compartmentalization in tissues as showed by other studies [53,54]. As a consequence, our observations during acute phase of infection may not be representative of the situations of individuals infected after mucosal exposure. However, after establishment of systemic infection we may consider that the compartmentalization of virus in cell subsets is probably weakly influenced by initial route of transmission.

## Conclusion

In conclusion, the levels of viral DNA and 2LTR circles in PBMCs measured very early in primary infection and/or at the set point followed the same natural course as plasma viral RNA levels and were predictive of the long-term progression of SIV infection. During primary infection, viral replication in gut-associated lymphoid tissue was correlated with plasma viral load, whereas no such correlation was observed for viral replication in secondary lymph nodes and the spleen. During chronic infection, viral replication in peripheral blood occurs mostly in the central memory CD4+ T cells with lower levels of replication observed in naïve CD4+ T cells and no replication in monocytes.

## Methods

### Animals and viral inoculation

Twenty-six adult cynomolgus macaques (*Macaca fascicularis*) were imported from Mauritius, and each weighing 4 to 6 kg were used in this study. They were housed in single cages within level 3 biosafety facilities. All animals used in this study tested negative for SIV, simian T-lymphotropic virus, herpes B virus, filovirus, simian retrovirus 1, simian retrovirus 2 and measles at the start of the study. All experimental procedures were conducted according to European guidelines for animal care ("*Journal Officiel des Communautés Européennes*," L358, 18 December 1986). Animals were sedated with ketamine chlorhydrate (Rhone-Mérieux, Lyons, France) before handling. Six macaques were inoculated intravenously (IV) with 50 times the 50% animal infectious dose of virus (50 AID_50_) of pathogenic SIVmac251 and six other animals received IV 5,000 AID_50 _of the same virus stock. These twelve macaques have been divided into two groups of six animals accordingly to their plasma viral load at set point (day 100 pi). For the exploration of viral dissemination in organs during primary infection, we inoculated the other group of 14 macaques intravenously with 50 AID_50 _of the same virus stock. These macaques were then euthanized on day 14 pi (n = 4), day 21 (n = 4), day 28 (n = 3) or day 100 (n = 3). We analysed the following organs: blood (plasma and PBMC), spleen, peripheral and mesenteric lymph nodes, ileum and rectum.

### SIVmac251 challenge stock

Cell-free virus stock of pathogenic SIVmac251 was kindly provided by A. M Aubertin (Université Louis Pasteur, Strasbourg, France). The virions were obtained from the cell-free supernatant of infected rhesus peripheral blood (PBMC). Cells were infected in vitro with a culture supernatant obtained from a co-culture of rhesus PBMC and a spleen homogenate from a rhesus macaque infected with SIVmac251 (provided by R. C. Desrosiers, New England Regional Primate Center, Southborough, Mass.).

### Virological and immunological measurements and tissue collection

Plasma and cell-associated viral loads as well as T-lymphocyte subsets were determined as previously described [19,55]. Immediately after the animals were euthanized, tissue samples (50 to 150 mg) were collected in quadruplicate from the spleen, peripheral lymph nodes (inguinal or axillary), mesenteric lymph nodes, ileum, and rectum and stored at -80°C.

### Phenotype and cell sorting of T cells and monocyte/macrophages

Naive, central memory and effector memory lymphocyte subsets and CD14+ monocytes from PBMCs were phenotyped with an LSRII analyser (BD Biosciences) or live sorted with a FACS ARIA machine (BD Biosciences). The cell sorting was performed twice from each animal. The following antibodies were used: CD3 Alexa Fluor 700 (clone SP34-2; BD Biosciences), CD4 PerCP (clone L200; BD Biosciences), CD8 FITC (clone DK25; DakoCytomation), CD28 PEcy7 (clone 28.2; BD Biosciences), CD95 APC (clone DX2; BD Biosciences) and CD14 PE (clone M5E2, BD Biosciences). CD4+CD28+CD95- cells were considered to be naive T cells, CD4+CD28+CD95+ cells were considered to be central memory cells and CD4+CD28-CD95+ cells were considered to be effector memory cells, as previously described (4). CD14+ cells were considered to be CD14+ monocytes. Stained cells were washed twice in PBS and were analysed by simultaneous four-way sorting on a FACS ARIA machine. The purity of isolated cells was analysed by flow cytometry. FlowJo software (TreeStar, Ashland, OR) was used for data analysis.

### Nucleic acid extraction

#### Tissue RNA and DNA extraction

Tissue lysates were obtained by the mechanical disruption of tissue samples in RA1 buffer (Macherey Nagel, Hoerdt, France) with a Precellys system, using 18 CK tubes with ceramic beads (Bertin Technologies, Montigny-le-Bretonneux, France). The tissue lysate was then diluted to 30 mg/ml in RA1, aliquoted and stored at -80°C until extraction. Total RNA was extracted in duplicate from aliquots of lysate, with the Nucleospin 96 RNA kit (Macherey Nagel). Contaminating DNA was removed from RNA samples by DNA elution and DNase treatment. Total DNA was recovered from tissue lysate with the Nucleospin 96 tissue kit (Macherey Nagel), according to the manufacturer's instructions.

#### RNA and DNA extraction from sorted cells

We collected 20,000 cells from each cell subpopulation directly after sorting in RA1 lysis buffer from the NucleoSpin^® ^RNAXS kit (MACHERY-NAGEL). Purified cell lysates from each subpopulation were split in half (lysate from ≈10,000 cells in each half), with one half used for RNA extraction with the NucleoSpin^® ^RNAXS kit and the other half used for DNA extraction with the NucleoSpin^® ^Tissue XS kit (MACHERY-NAGEL). All extractions were performed according to the manufacturer's instructions. The RNA or DNA was eluted in 40 μl of nuclease-free water and frozen immediately at -80°C for storage until analysis.

### Viral RNA quantification in tissues and sorted cells

RNA extracted from tissue or sorted cells was analysed in duplicate in an RT-qPCR assay with the Superscript III Platinum one-step quantitative RT-PCR system (Invitrogen, Cergy-Pontoise, France), using the SIV *gag *primers and probe described elsewhere [55]. The reaction was carried out and the data were acquired with the I-Cycler real-time PCR system (Biorad, Marnes-la-Coquette, France).

The probe and primers, described by Hofmann-Lehmann et al. [56], were designed to bind within the conserved SIV *gag *region, a marker of transcription of full length transcripts. The sequences of the primers used were: 5'-CAATTTTACCCAGGCATTTAATGTT-3' and 5'-GCAGAGGAGGAAATTACCCAGTAC-3' (nucleotide position 389-480). The TaqMan probe sequence was 5'-TGTCCACCTGCCATTAAGCCCGA-3', labeled at the 5' end with a fluorescence reporter dye, FAM (6-carboxyfluorescein), and at the 3' end with the quencher dye TAMRA (6-carboxytetramethyl-rhodamine).

#### Quantification of viral RNA in tissue

RNA input was normalized by simultaneously quantifying GAPDH RNA with a previously described primer set and probe [57]. We included negative controls and serial 10-fold dilutions of SIV and GAPDH RNA for each experiment, to assess amplification efficiency. As the efficiencies of all GAPDH and SIV reactions were similar, we conducted a 2^-ΔCt ^analysis. Results are expressed as number of SIV RNA copies/number of GAPDH RNA copies.

#### Quantification of viral RNA in sorted cells

Absolute numbers of copies of viral RNA were normalised to 10,000 cells and results are expressed as the number of SIV RNA copies per 10^6 ^cells. Total RNA was extracted from ≈10,000 sorted cells. We therefore checked the numbers of cells in each unknown sample. We generated RNA standards (serially diluted 1:10 (up to 10^-4^)) for 10,000 cells from uninfected macaques. The GADPH gene was then amplified simultaneously with a primer set and probe, as previously described (5). GAPDH-RNA levels in unknown samples were inferred by comparing threshold cycle (Ct) values against a calibration curve. Unknown samples had levels of amplifiable cDNA equivalent to those for 10,000 cells.

### Total viral DNA quantification in tissues and sorted cells

DNA extracted from tissues or sorted cells was analyzed in duplicate by a real-time PCR assay, with the Platinum qPCR SuperMix UDG kit (Invitrogen) and SIV *gag *primers and probe, as previously described [55]. The reaction was carried out and data were acquired and analysed with the I-Cycler real-time PCR system (Biorad). The number of copies of SIV DNA in unknown samples was inferred by plotting the threshold cycle (Ct) value against a calibration curve (gag SIVmac251 DNA plasmid, linear dynamic range 10 to 10^7 ^copies). The GAPDH gene was simultaneously amplified from genomic DNA, for normalisation, using a previously described primer set and probe [57]. Results are expressed as the number of copies of SIV DNA per 10^6 ^cells.

### SIV 2-LTR circle quantification in tissues and sorted cells

The 2-LTR junction (≈ 305 bp) was amplified in duplicate from tissue DNA or sorted cells, in a 25 μl reaction mixture consisting of 1× Platinium^® ^qPCR SuperMix-UDG (Invitrogen), 450 nM of each primer and 250 nM fluorogenic probe. The primers used for amplification were 2LTRs 5'-TAAGCTAGTGTGTGTTCCCAT-3' (21 bp) and REVN1 5'-CTCCTGTGCCTCATCTGATACA-3' (22 bp). The TaqMan probe sequence was 5'- [6~FAM]AGCCGCCGCCTGGTCAACTCG [TAMARA~6~FAM]-3' (21 bp). Amplifications were carried out and data acquired with an I-Cycler real-time PCR system (Biorad). We used the following PCR parameters: denaturation for 10 minutes at 95°C, followed by 50 cycles of 95°C for 10 s, 61°C for 10 s and 72°C for 20 s. The copy number of 2-LTR circles was determined from a standard curve generated by the PCR amplification of serial dilutions of the PCR4TOPO2-LTR plasmid including the SIVmac251 2-LTR junction. The GAPDH gene was amplified from genomic DNA, in parallel. Results are expressed as the number of copies of the SIV 2LTR sequence per 10^6 ^cells.

### In situ hybridization

Cells expressing SIV RNA in lymphoid tissues were identified by *in situ *hybridization of sections of fixed tissues. Radioactive *in situ *hybridization was performed as previously described [58]. The specificity of the hybridization signal was systematically checked by hybridizing sense probes on successive sections. Slides were counterstained with Mayer's hemalun and mounted in permanent mounting media (Dako). Image acquisition and analysis were performed on a Nikon i90 photomicroscope using NIS-elements software.

### Statistical analysis

Non-parametric Spearman's rank correlation test was used to investigate the correlation between 2-LTR circle levels and total viral DNA or plasma viral RNA levels in longitudinal analysis. The Mann-Whitney test was used to compare the levels of viral RNA, 2-LTR circles and total viral DNA of different groups of macaques. Statistical analysis was carried out with Statview software (SAS Institute, Inc., Cary, N.C).

## Competing interests

The authors declare that they have no competing interests.

## Authors' contributions

Conceived and designed the experiments: RLG, PR. Performed the experiments: AM, OB, PS, BD, PB, TA, IK, PR, RLG. Analyzed the data: AM, OB, PS, PR, BV, RLG. Wrote the paper: AM, PS, OB, RLG, PR.

## Supplementary Material

Additional file 1 **Viral dissemination in the thymus of macaques during primary infection with SIVmac251**. The viral DNA was evaluated in thymus tissue from macaques infected with SIVmac251, on days 14, 21 and 28. Absolute copy numbers for viral DNA were calculated to the GAPDH and normalized to one million of cells. Click here for file
