# Supplementary material for: Dynamics of viral replication in blood and lymphoid tissues during SIVmac251 infection of macaques
Source: Retrovirology. 2009 Nov 23;6:106. doi: 10.1186/1742-4690-6-106 (PMC2789052; doi:10.1186/1742-4690-6-106)
Supplement: Additional file 1 — Viral dissemination in the thymus of macaques during primary infection with SIVmac251. The viral DNA was evaluated in thymus tissue from macaques infected with SIVmac251, on days 14, 21 and 28. Absolute copy numbers for viral DNA were calculated to the GAPDH and normalized to one million of cells. [file 1742-4690-6-106-S1.PPT]

## Slide 1
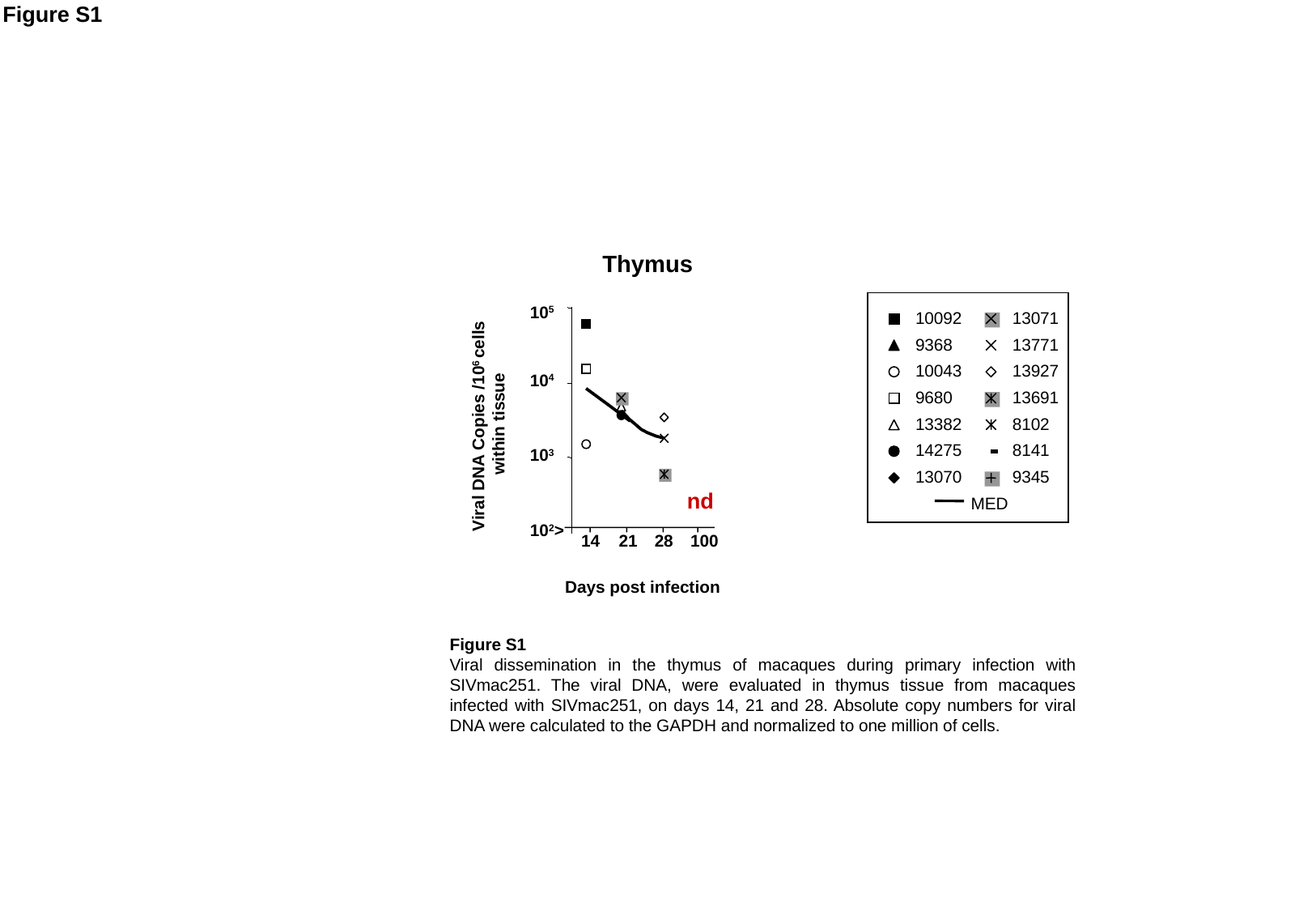

Figure S1
Thymus
105
10092
13071
13771
9368
10043
13927
104
9680
13691
Viral DNA Copies /106 cells
within tissue
8102
13382
14275
8141
103
13070
9345
nd
MED
102>
14
21
28
100
Days post infection
Figure S1
Viral dissemination in the thymus of macaques during primary infection with SIVmac251. The viral DNA, were evaluated in thymus tissue from macaques infected with SIVmac251, on days 14, 21 and 28. Absolute copy numbers for viral DNA were calculated to the GAPDH and normalized to one million of cells.
